# CROSSING THE DIGITAL DIVIDE: ASSESSING DIGITAL READINESS AMONG UNDERSERVED OLDER ADULTS

**DOI:** 10.1093/geroni/igad104.1718

**Published:** 2023-12-21

**Authors:** Sheila Salinas Navarro, Mengzhao Yan, Sindy Lomeli, Kathleen Wilber

**Affiliations:** University of Southern California, Los Angeles, California, United States; University of Southern California, Los Angeles, California, United States; University of Southern California, Los Angeles, California, United States; University of Southern California, Los Angeles, California, United States

## Abstract

The California Department of Aging launched the “Connecting, Health, Aging, & Technology (CHAT) Program, “aimed at bridging the digital divide and mitigating social isolation among older adults by providing an iPad device and technology training. Because providing a device does not guarantee uptake, we examined factors associated with technology adoption among research participants (n=1,219). We used a global “digital readiness” scale, and two open-ended questions to examine potential device uptake. We ran multivariate regressions with explanatory independent variables including demographic characteristics (age, gender, race/ethnicity, living arrangement, and education), health indicators (self-rated health and mental health status) and the intention to adopt technology against the global readiness question. The analysis showed that digital readiness was associated with age and education, and Asian or Pacific Islander older adults were more ready than non-Hispanic Whites. Further, digital readiness was negatively associated with mental health but positively associated with self-rated health. Digital readiness was also associated with an increased intention to adopt technology. A content analysis of the open-ended questions in Atlas.ti informed the regression analyses and revealed participants’ strong desire for digital self-efficacy, informed citizenship, lifelong learning, a proactive desire to improve quality of life, and the desire to foster connection to family and friends. Our research highlights the personal and multifaceted nature of technology adoption among older adults, and the complexity of fostering digital inclusion on a large scale.

